# Income and Severe Hypoglycemia in Type 2 Diabetes

**DOI:** 10.1001/jamanetworkopen.2025.13293

**Published:** 2025-06-02

**Authors:** Misook Kim, Kyungdo Han, Kyuna Lee, Bongseong Kim, Kyuho Kim, Seung-Hyun Ko, Yu-Bae Ahn, Seung Yeon Kim, Seung-Hwan Lee, Jae-Seung Yun

**Affiliations:** 1 Division of Endocrinology and Metabolism, Department of Internal Medicine, Seoul St Mary’s Hospital, College of Medicine, The Catholic University of Korea, Seoul, Republic of Korea; 2 Department of Statistics and Actuarial Science, Soongsil University, Seoul, Republic of Korea; 3 Department of Biomedicine and Health Science, The Catholic University of Korea, Seoul, Korea; 4 Division of Endocrinology and Metabolism, Department of Internal Medicine, St Vincent’s Hospital, College of Medicine, The Catholic University of Korea, Seoul, Republic of Korea

## Abstract

**Importance:**

Socioeconomic disparities are increasingly recognized as key factors in health outcomes among patients with type 2 diabetes. Understanding how income level and its changes are associated with severe hypoglycemia risk may inform targeted interventions and policy decisions.

**Objective:**

To evaluate the association between income level, including changes in income status, and the risk of severe hypoglycemia, and to assess whether this association varies across key subgroups in middle-aged adults.

**Design, Setting, and Participants:**

This retrospective cohort study included adults aged 40 to 70 years with type 2 diabetes from the Korean National Health Information Database (NHID) who underwent health examinations from 2015 to 2016. Participants aged 40 to 70 years with type 2 diabetes and valid income information were recruited between 2006 and 2010 from the UK Biobank (UKBB) and were included for sensitivity analysis. Data were analyzed from January 2023 to September 2024.

**Exposures:**

Participants were categorized into annual income quartiles (NHID) or self-reported income brackets (UKBB). In the NHID, medical aid beneficiaries (ie, those receiving government medical aid due to income below 40% of the median income and limited assets) were identified, and income changes over a 5-year period were assessed.

**Main Outcomes and Measures:**

Severe hypoglycemia requiring medical intervention.

**Results:**

Among 1 838 362 adults with type 2 diabetes from the NHID (mean [SD] age, 57.1 [8.1] years; 1 157 263 [63.0%] male) and 17 287 participants from the UKBB (mean [SD] age, 56.9 [6.8] years; 11 522 [66.7%] male), lower income was associated with a higher risk of severe hypoglycemia (NHID: hazard ratio [HR], 2.50; 95% CI, 2.33-2.57; UKBB: HR, 5.38; 95% CI, 1.72-16.85). In the NHID, individuals whose income increased from the lowest quartile or medical aid status to the fourth quartile over 5 years had a significantly lower risk (HR, 0.74; 95% CI, 0.67-0.81; *P* for trend <.001), whereas receiving medical aid for at least 1 year was associated with a significantly higher risk (HR, 1.71; 95% CI, 1.54-1.89). The association between income and severe hypoglycemia was consistently greater in men, individuals not using insulin, those without chronic kidney disease, and those with a shorter duration of diabetes.

**Conclusions and Relevance:**

In this cohort study of adults with type 2 diabetes, low income was found to be a significant factor associated with risk for severe hypoglycemia. Addressing socioeconomic disparities and implementing targeted interventions may help reduce the incidence and severity of hypoglycemia.

## Key Points

**Question:**

Are low income and changes in income level associated with the risk of severe hypoglycemia in individuals with type 2 diabetes?

**Findings:**

In this cohort study of 1.84 million individuals in Korea and 17 287 in the UK, lower income was associated with significantly higher risk of severe hypoglycemia; prolonged low income was associated with increased risk, while income improvements were associated with reduced risk. Men had consistently higher risks compared with women.

**Meaning:**

These findings suggest that addressing socioeconomic disparities and improving financial stability in low-income populations are critical to reducing the risk of severe hypoglycemia and improving diabetes care.

## Teaser

This cohort study evaluates the association between income level and severe hypoglycemia in adults with type 2 diabetes from Korea.

## Introduction

Managing the risk of hypoglycemia is a critical component of improving patient care in type 2 diabetes.^1,2,3^ Severe hypoglycemia, a major diabetes complication, poses significant risks, including increased diabetes-related mortality, morbidity, and heightened medical costs.^4,5^ Understanding the factors that contribute to severe hypoglycemia is essential for developing targeted interventions.

The relationship between socioeconomic status and major health outcomes is well established, particularly concerning chronic diseases such as type 2 diabetes.^6,7,8^ With increasing global disparity, there is growing interest in social determinants of health, including socioeconomic disparities.^9^ Having a low income is often associated with limited health care access, poor medication adherence, inadequate nutrition, and more comorbidities, all of which may contribute to increased risk of severe hypoglycemia.^10^ Previous studies have reported that food insecurity, underinsurance, homelessness, and low annual household income are associated with increased risk of severe hypoglycemia.^11,12,13^ The American Diabetes Association standards of care for diabetes highlight socioeconomic status as a significant risk factor for hypoglycemia, alongside clinical, biological, social, cultural, economic, and other risk factors.^1^ While previous studies have examined this association, few have specifically investigated the cumulative duration of low income or the impact of income fluctuations on hypoglycemia risk.

This study aims to investigate the association between income level and the risk of severe hypoglycemia in individuals with type 2 diabetes using a large nationwide cohort. Specifically, we examine whether baseline income, income stability, and income changes over time influence severe hypoglycemia risk. To enhance the generalizability and robustness of our findings, we conducted a complementary analysis using the UK Biobank dataset and performed subgroup analyses stratified by sex, obesity status, insulin use, chronic kidney disease, and diabetes duration to assess potential effect modifications in this association.

## Methods

### Study Population and Design

The study population was selected from the National Health Information Database (NHID) and UK Biobank (UKBB) databases, with specific inclusion and exclusion criteria applied to each dataset. For the NHID dataset, individuals diagnosed with type 2 diabetes (T2D) who underwent health examinations from 2015 to 2016 were included. Exclusion criteria included individuals outside the defined middle-aged range (40-70 years), those with missing income data during the 5-year evaluation period, and those with incomplete information on key covariates for analysis. For the UKBB dataset, individuals aged 40 to 70 years diagnosed with T2D from 2006 to 2010 were included if income information was available, while those with missing income data were excluded. After applying these inclusion and exclusion criteria, the final study population was determined. Detailed information on the participant selection criteria is provided in eFigure 1 in Supplement 1; coverage of population, self-reported ethnicity, and income assessment methods are described in the eMethods in Supplement 1.

This study was approved by the institutional review board of Soongsil University and complies with the ethical guidelines of the Declaration of Helsinki of the World Medical Association. Participants of the NHID data did not provide informed consent as data were deidentified. UKBB participants provided written informed consent for their data to be collected, stored, and used for health-related research. Identifiable information is separated from the main dataset, and researchers only have access to deidentified data. This study was conducted following the Strengthening the Reporting of Observational Studies in Epidemiology (STROBE) reporting guidelines.

### Definition of Income Category and Severe Hypoglycemia

In the NHID, T2D was defined using a combination of a diabetes diagnosis code, fasting glucose level, and the use of diabetes medication. In contrast, the UKBB defined T2D through a combination of questionnaire responses, diagnosis codes, HbA_1c_ level, and the use of diabetes medication.

The National Health Insurance Service (NHIS) in South Korea charges health insurance premiums proportionate to monthly household income, which serves as a proxy for income. Income status was categorized into 4 levels, from the first to the fourth quartile for each year, with an additional classification for medical benefit beneficiaries representing the lowest 3% income group. Medical aid recipients were defined as those receiving medical aid (ie, individuals receiving government medical aid due to income below 40% of the median income and limited assets) in any given year. Income level classification in the NHID cohort is detailed in the eMethods in Supplement 1. In the NHID, income group levels were calculated annually from 2012 to 2016, considering income ranges from each index year through the preceding 5 years. To investigate the association between prolonged low-income status and risk of severe hypoglycemia, cumulative years of low-income status were calculated, and changes in individual income were followed up (eFigure 2 in Supplement 1). Severe hypoglycemia, the primary outcome, was defined as any event occurring by December 31, 2022, based on the diagnosis code. Follow-up for each participant ended at the earliest occurrence of one of the following events: December 31, 2022; death; or the first diagnosis of severe hypoglycemia after the follow-up start date.

In the UKBB, income information was collected via questionnaire and categorized into the following brackets: less than £18 000 per year, £18 000 to £29 999 per year, £30 000 to £51 999 per year, £52 000 to £100 000 per year, and more than £100 000 per year. Severe hypoglycemia in the UKBB was also defined as hospitalization with hypoglycemia in participants with diabetes, identified using diagnosis codes. Follow-up for each participant ended at the earliest occurrence of one of the following events: April 30, 2021, death, or the first diagnosis of severe hypoglycemia after the follow-up start date.

### Variable Measurement

Ethnicity-appropriate World Health Organization body mass index (BMI) criteria for obesity were applied for Asian and non-Asian participants from the NHID and UKBB cohorts.^14^ Lifestyle information for both cohorts was collected based on surveys. Smoking status was categorized as current smoker or nonsmoker. The NHID classified alcohol consumption into 3 groups based on daily intake according to the US Dietary Guidelines: nondrinkers (0 g of alcohol per day), light drinkers (less than 30 g of alcohol per day), and heavy drinkers (more than 30 g of alcohol per day). In the UKBB, individuals who reported not drinking alcohol at all or drinking only on special occasions were categorized as nondrinkers, those who reported consuming alcohol more than 5 times a week were categorized as heavy drinkers, and the remaining responses were categorized as light drinkers. Fasting glucose level was used as an indicator of glycemic status in the NHID, as HbA_1c_ data were not available, while HbA1c was used in the UKBB due to the unavailability of fasting glucose level. Disease conditions, including T2D, hypertension, dyslipidemia, cardiovascular disease (CVD), chronic kidney disease (CKD), and a history of severe hypoglycemia, were defined based on diagnosis codes, medication information, and laboratory results. In the NHID, household size was defined based on NHIS family member records, while in the UKBB, it was identified through a touchscreen survey question regarding the number in the household. Main variable definitions are summarized in eTables 1 and 2 in Supplement 2.

### Statistical Analysis

Categorical variables were reported as percentages, while continuous variables were reported as mean (SD) or median (IQR), depending on normality assessment. The normality of continuous variables was evaluated using the Kolmogorov-Smirnov test. Given that triglyceride levels followed a log-normal distribution, they were reported as geometric mean (95% CI) and compared using the Wilcoxon rank-sum test. For other nonnormally distributed continuous variables, the Mann-Whitney U test was used to assess between-group differences. Normally distributed continuous variables were compared using the *t* test, and categorical variables were analyzed using the χ^2^ test. The association between income status and severe hypoglycemia was assessed using multivariate Cox proportional hazards regression models, with results presented as hazard ratios (HRs) with 95% CIs. Regression analyses were adjusted for potential confounding factors and/or interventions, including age, sex, race (UKBB only), smoking, alcohol consumption, physical activity, BMI, hypertension, dyslipidemia, CVD, CKD, diabetes duration, and history of severe hypoglycemia. Household size, a potential confounder influencing income and hypoglycemia risk, was included as an adjustment variable in the multivariate models.

The NHID data allowed for the assessment of income changes over 5 years, enabling analysis of the cumulative outcome of income duration on risk of severe hypoglycemia. The cumulative duration outcome of income was defined as the sum of the periods included in the relevant income bracket, based on 5 years of health insurance contributions. Additionally, changes in income status in the first and last years and occurrence of severe hypoglycemia were identified in the NHID. Subgroup analysis for effect modification was performed in both NHID and UKBB cohorts, considering baseline gender, obesity, duration of diabetes, insulin use, and CKD. In the UKBB, where the sample size was smaller, diabetes duration was analyzed in 2 groups: less than 5 years and more than 5 years. The proportion of missing data for each key variable is summarized in eTable 3 in Supplement 2. Information on missing data handling is provided in eMethods in Supplement 1. Statistical analyses were performed using SAS version 9.4 (SAS Institute) and *R* version 4.2.0 (R Project for Statistical Computing). The significance level was set at a 2-sided *P* value of .05, with values below this threshold considered statistically significant. Data were analyzed from January 2023 to September 2024.

## Results

### Baseline Characteristics and Income Levels

A total of 1 838 362 participants from the NHID were included in the analysis, with a median (IQR) follow-up duration of 6.7 (6.2-7.2) years. The Table presents participant baseline characteristics stratified by baseline income level. In the NHID cohort, the mean (SD) age at the index date was 57.1 (8.1) years, and 1 157 263 participants (63.0%) were male. Additionally, 54 784 individuals (3.0%) were classified as medical aid beneficiaries. Analysis indicated that lower income was associated with a higher proportion of women and of heavy drinkers. Additionally, low-income groups exhibited a poor metabolic profile and a higher number of comorbidities. Baseline characteristics were further examined according to the duration of receiving medical aid. Out of a total of 1 838 362 patients with T2D, 67 835 (3.7%) were identified as low income (recipients of medical aid) for at least 1 year within a 5-year period. Among these, 38 530 (2.1%) continuously received low-income medical aid. As the duration of medical aid receipt increased, the proportion of women and nonsmokers rose, while the proportion of individuals with heavy alcohol consumption declined. While there was a high prevalence of obesity class II, an increase in the proportion of underweight participants was also noted. Additionally, the rate of low HDL-cholesterol tended to decrease alongside increases in medical aid duration, frequency of insulin use, and prevalence of CKD. Finally, the history of severe hypoglycemia also increased among the bottom 25% of income earners, similar to those receiving medical aid (eTables 4 and 5 in Supplement 2).

**Table.** Baseline Characteristics of the Korean National Health Information Database Study Population by Income Group in the Last Year of the Index Period

Characteristic	Patients, No. (%)						*P* value for trend
	Total (N = 1 838 362)	Medical aids (n = 54 784)	Income group for the last year of the index period, quartile				
			1 (n = 372 678)	2 (n = 366 434)	3 (n = 460 475)	4 (n = 583 991)	
Age, mean (SD), y	57.1 (8.1)	56.4 (6.8)	57.9 (7.8)	57.0 (7.8)	56.9 (8.3)	57.0 (8.3)	<.001
Sex							
Male	1 157 263 (63.0)	29 052 (53.0)	207 080 (55.6)	227 590 (62.1)	298 443 (64.8)	395 098 (67.7)	<.001
Female	681 099 (37.0)	25 732 (47.0)	165 598 (44.4)	138 844 (37.9)	162 032 (35.2)	188 893 (32.3)	
Smoking							
Nonsmoker	936 627 (51.0)	29 000 (53.0)	206 880 (55.1)	187 120 (51.1)	228 179 (49.6)	285 448 (48.9)	<.001
Ex-smoker	440 082 (23.9)	8431 (15.4)	75 770 (20.3)	82 707 (22.6)	112 490 (24.4)	160 684 (27.5)	
Current smoker	461 653 (25.1)	17 353 (31.7)	90 028 (24.2)	96 607 (26.4)	119 806 (26.0)	137 859 (23.6)	
Drinking^a^							
None	983 103 (53.5)	39 427 (72.0)	216 424 (58.1)	195 886 (53.5)	241 321 (52.4)	290 045 (49.7)	<.001
Mild	666 091 (36.2)	11 439 (20.9)	122 467 (32.9)	132 846 (36.3)	169 062 (36.7)	230 277 (39.4)	
Heavy	189 168 (10.3)	3918 (7.2)	33 787 (9.1)	37 702 (10.3)	50 092 (10.9)	63 669 (10.9)	
Household No., median (IQR)	3 (2-4)	1 (1-2)	2 (1-3)	2 (1-3)	3 (2-4)	3 (2-5)	<.001
Insulin user	165 180 (9.0)	11 243 (20.5)	33 009 (8.9)	32 040 (8.7)	40 020 (8.7)	48 868 (8.4)	<.001
Duration of diabetes							
New onset	215 447 (11.7)	3557 (6.49)	43 830 (11.8)	46 271 (12.6)	57 420 (12.5)	64 369 (11.0)	<.001
<5 y	804 201 (43.7)	20 791 (38.0)	160 772 (43.1)	161 706 (44.1)	203 376 (44.2)	257 556 (44.1)	
<10 y	392 553 (21.4)	16 761 (30.6)	79 647 (21.4)	76 001 (20.7)	95 810 (20.8)	124 334 (21.3)	
≥10 y	426 161 (23.2)	13 675 (25.0)	88 429 (23.7)	82 456 (22.5)	103 869 (22.6)	137 732 (23.6)	
Previous SH	22 134 (1.2)	2448 (4.5)	4536 (1.2)	4061 (1.11)	5291 (1.2)	5798 (1.0)	<.001
Fasting glucose, median (IQR), mg/dL	135 (122-158)	135 (116-161)	135 (116-162)	134 (115-163)	133 (116-161)	132 (112-159)	<.001
BMI^b^	25.4** (**3.5)	25.4** (**4.2)	25.3** (**3.5)	25.3** (**3.5)	25.4** (**3.5)	25.5** (**3.3)	<.001
Obesity category, BMI							
Underweight, <18.5	18 926 (1.07)	1213 (3.2)	149 (2.7)	155 (2.8)	193 (2.8)	280 (2.5)	<.001
Normal weight, 18.5-22.9	395 774 (22.4)	9646 (25.0)	1420 (25.9)	1494 (26.5)	1768 (26.0)	2945 (25.9)	
Overweight, 23.0-24.9	431 768 (24.4)	7620 (19.8)	1115 (20.3)	1198 (21.3)	1430 (21.0)	2468 (21.7)	
Obesity class I, 25.0-29.9	761 915 (43.0)	14 671 (38.1)	2085 (38.00)	2128 (37.8)	2600 (38.2)	4369 (38.4)	
Obesity class II, ≥30.0	162 144 (9.7)	5380 (14.0)	719 (13.1)	659 (11.7)	814 (12.0)	1316 (11.6)	
Obesity	924 059 (52.2)	20 051 (52.0)	2804 (51.1)	2787 (49.5)	3414 (50.2)	5685 (50.0)	<.001
Waist circumference, mean (SD), cm	86.1** (**8.9)	86.5** (**10.4)	85.7** (**9.0)	85.7** (**8.9)	86.2** (**8.8)	86.4** (**8.6)	<.001
Abdominal obesity	723 934 (40.9)	12 021 (31.2)	1816 (33.1)	1728 (30.7)	2093 (30.8)	3610 (31.7)	<.001
Hypertension	1 017 251 (55.3)	34 037 (62.1)	210 049 (56.4)	204 090 (55.7)	256 703 (55.8)	312 372 (53.5)	<.001
Systolic blood pressure, mean (SD), mm Hg	127.7** (**14.8)	125.9** (**15.8)	127.9** (**15.0)	128.1** (**15.0)	128.2** (**14.8)	127.2** (**14.4)	<.001
Diastolic blood pressure, mean (SD), mm Hg	78.4** (**9.9)	77.3** (**10.2)	78.1** (**9.9)	78.6** (**10.0)	78.7** (**9.9)	78.2** (**9.7)	<.001
Dyslipidemia	1 074 429 (58.4)	35 652 (65.1)	218 979 (58.8)	208 134 (56.8)	266 094 (57.8)	345 570 (59.2)	<.001
Total cholesterol, median (IQR), mg/dL	183 (154-214)	178 (150-209)	177 (149-209)	176 (149-210)	177 (148-208)	174 (147-206)	<.001
Triglyceride, geometric mean (95% CI), mg/dL	139 (139-139)	141 (140-141)	137 (137-138)	138 (138-138)	140 (140-141)	139 (138-139)	<.001
HDL cholesterol, median (IQR), mg/dL	50 (42-59)	48 (39-57)	50 (42-60)	50 (43-59)	49 (41-58)	48 (40-58)	<.001
LDL cholesterol, median (IQR), mg/dL	100 (75-128)	96 (71-123)	95 (71-123)	93 (70-122)	94 (70-122)	93 (70-121)	<.001
Chronic kidney disease	133 641 (7.3)	7042 (12.9)	29 632 (8.0)	25 377 (6.9)	32 624 (7.1)	38 966 (6.7)	<.001
Estimated GFR, median (IQR), ml/min/1.73m^2^	88.1 (74.8-103.6)	87.2 (72.2-105.3)	86.8 (71.7-105.7)	85.6 (71.7-105.0)	84.6 (71.7-105.0)	83.5 (70.0-104.3)	<.001

Abbreviations: BMI, body mass index; GFR, glomerular filtration rate; HDL, high-density lipoprotein; LDL, low-density lipoprotein; SH, severe hypoglycemia.

SI conversion factor: To convert HDL to mmol/L, multiply by 0.0259; LDL to mmol/L, multiply by 0.0259; total cholesterol to mmol/L, multiply by 0.0259; triglycerides to mmol/L, multiply by 0.0113.

^a^ The NHID classified alcohol consumption into 3 groups based on daily intake according to the US dietary guidelines: nondrinkers (0 g of alcohol per day), light drinkers (less than 30 g of alcohol per day), and heavy drinkers (more than 30 g of alcohol per day).

^b^ Calculated as weight in kilograms divided by height in meters squared.

eTable 6 in Supplement 2 highlights the baseline characteristics of the top 5% income earners. Within the study population, 201 122 individuals (10.9%) were in the top 5% income bracket at least once over 5 years, with 56 318 (3.1%) maintaining high-income status throughout the entire period. Longer high-income duration was associated with a higher proportion of men, increased prevalence of obesity (BMI 25.0-30.0; calculated as weight in kilograms divided by height in meters squared), and lower prevalence of both obesity class II and underweight status. Prolonged high income was also associated with better metabolic profile, reduced insulin use, lower prevalence of CKD, and fewer previous severe hypoglycemia events. Participants within the top 25% income bracket exhibited similar patterns to those in the top 5% income bracket (eTables 6 and 7 in Supplement 2).

### Analysis of Severe Hypoglycemia Risk Based on Baseline Income Level, Income Level Changes, and Duration

**Figure 1.**
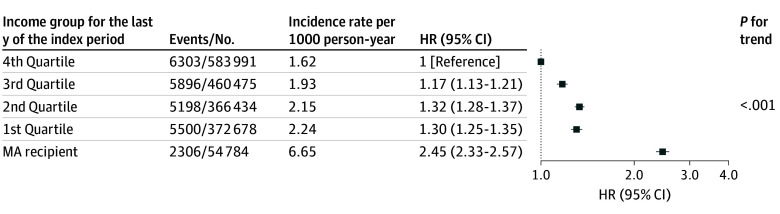
Association Between Low Income and Risk of Severe Hypoglycemia by Income Group in the Last Year of the Index Period HR indicates hazard ratio; MA, medical aid (ie, individuals receiving government medical aid due to income below 40% of the median income and limited assets).

**Figure 2.**
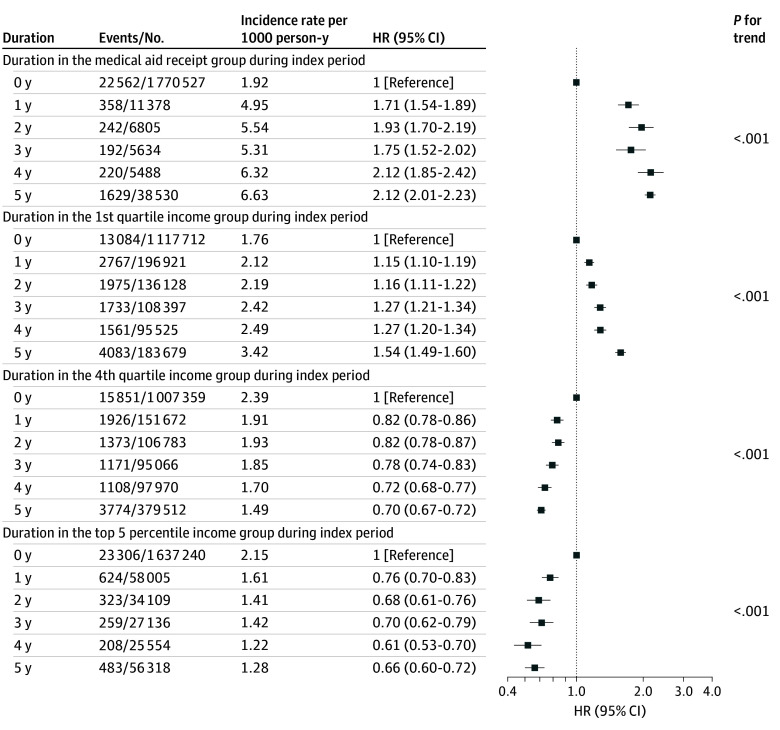
Severe Hypoglycemia Outcomes by Duration in Income Group During the Index Period

Figure 1 displays the association between the risk of severe hypoglycemia and baseline income at the index date. When compared with income quartile Q4, risk of severe hypoglycemia significantly increased as income decreased, starting from quartile Q3, with the highest risk observed among medical aid recipients (HR, 2.45; 95% CI, 2.33-2.57; *P* < .001). In model 6, adjusting for household size did not alter the association between income and the risk of severe hypoglycemia (eTable 8 in Supplement 2). Figure 2 presents the risk of severe hypoglycemia in association with the duration in the top 5% income bracket or receiving medical aid over a 5-year period. Even after adjusting for various income-related confounding variables, the risk of severe hypoglycemia in the group that had been aid beneficiaries for more than 1 year was nearly double that of the group that had not received aid (eTable 9 in Supplement 2). However, risk of severe hypoglycemia did not show a dose-dependent pattern based on the duration of being a medical aid beneficiary; rather, the risk significantly increased if the patient had been a beneficiary at any point. Among individuals in the top 5% high-income group, the risk of severe hypoglycemia decreased for those who continuously remained in that bracket. (eTable 9 in Supplement 2). The cumulative incidence of severe hypoglycemia stratified by income duration and baseline income level is presented in eFigure 3 in Supplement 1.

We further elucidated the risk of severe hypoglycemia according to change in income group after 5 years among low-income individuals whose income in the first year was in the bottom 25% or who were recipients of medical aid (eTable 10 in Supplement 2). The risk of severe hypoglycemia was substantially reduced in participants whose income increased compared with those who remained in the low-income group for the entire 5 years. Individuals whose income increased from the lowest quartile or medical aid status to the fourth quartile over 5 years had a significantly lower risk (HR, 0.74; 95% CI 0.67-0.81; *P* for trend <.001), whereas receiving medical aid for at least 1 year was associated with a 1.7-fold higher risk (HR, 1.71; 95% CI, 1.54-1.89).

### Subgroup Analysis Based on Baseline Income Level

**Figure 3.**
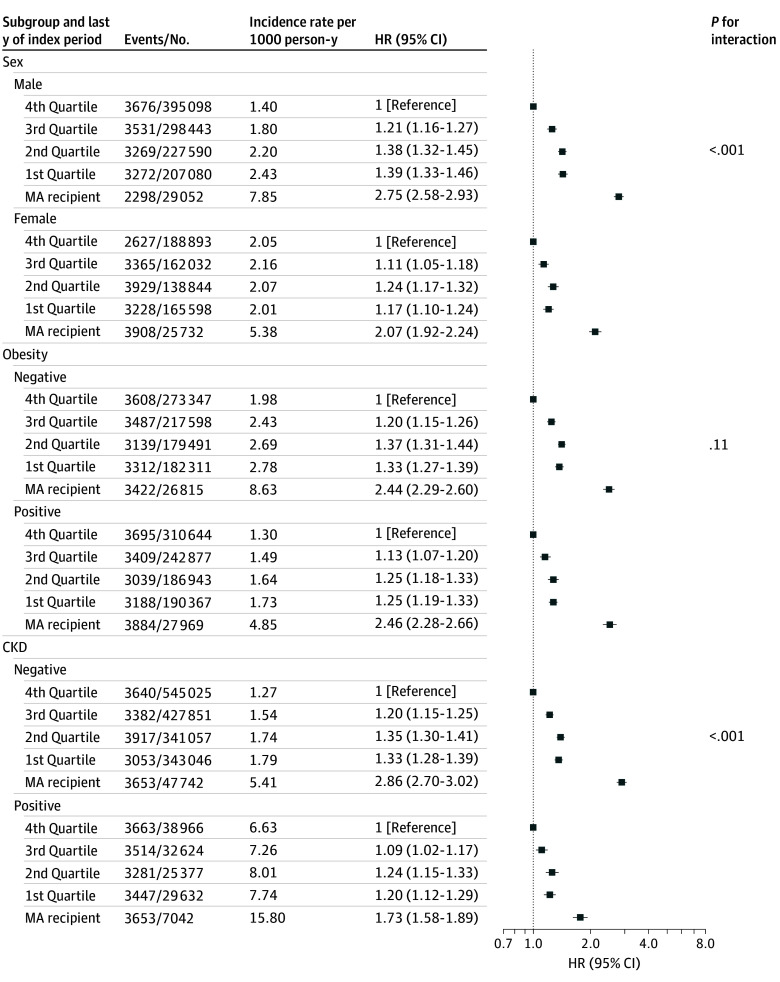
Subgroup Analysis by Sex, Obesity, and Chronic Kidney Disease (CKD) of the Association Between Income Group in the Last Year of the Index Period and Severe Hypoglycemia Outcomes MA indicates medical aid (ie, individuals receiving government medical aid due to income below 40% of the median income and limited assets).

**Figure 4.**
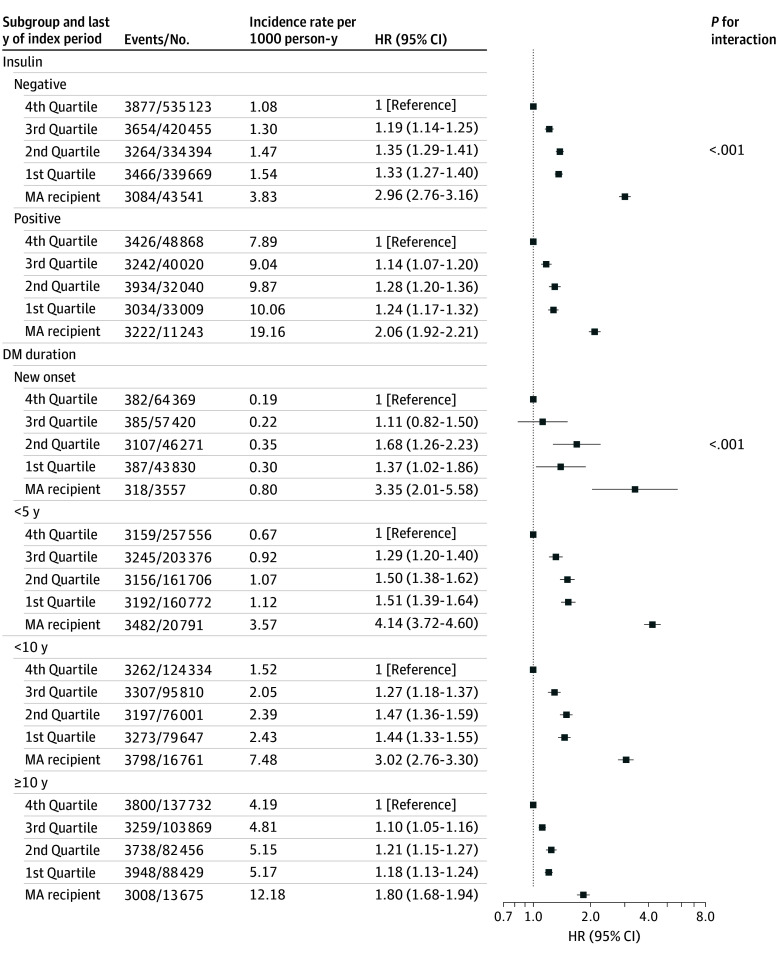
Subgroup Analysis by Insulin Use and Diabetes Duration of the Association Between Income Group in the Last Year of the Index Period and Severe Hypoglycemia Outcomes MA indicates medical aid (ie, individuals receiving government medical aid due to income below 40% of the median income and limited assets).

Subgroup analysis confirmed the association between baseline income and severe hypoglycemia, as illustrated in Figures 3 and 4. Among low-income groups, the association between lower income and increased risk of severe hypoglycemia was more pronounced in men (eTable 11 in Supplement 2). Additionally, this association was more evident in participants with a shorter duration of diabetes, and in aid beneficiaries without CKD (eTable 12 in Supplement 2). For the top 5% of high income earners, decreased risk of severe hypoglycemia with higher income was consistently observed across all subgroups, irrespective of gender, diabetes duration, insulin use, or CKD status.

### Sensitivity Analysis in UKBB Participants

We also examined the association between income and severe hypoglycemia in participants from the UKBB. A total of 17 287 participants from the UKBB with T2D were included in the analysis, with a mean (SD) age of 59.6 (6.8) years, a median (IQR) follow-up duration of 12.1 (11.4-12.9) years, and 11 522 (66.7%) were male. Among them, 6461 individuals (37.4%) had low incomes (£18 000 or less). Similar to the baseline characteristics observed in the NHID dataset, UKBB participants with lower income had a higher proportion of women, smokers, individuals with abdominal obesity, and individuals with obesity. They also had a higher prevalence of insulin use, hypertension, and dyslipidemia (eTable 13 in Supplement 2). Furthermore, in this second cohort, the risk of severe hypoglycemia again sequentially increased with decreasing income level. Even after adjusting for various variables, the lowest-income group had up to 5.38 times higher risk of severe hypoglycemia compared with the high-income group (HR, 5.38; 95% CI, 1.72-16.85) (eTable 14 in Supplement 2). Subgroup analysis revealed that the risk of severe hypoglycemia was significantly higher in low-income men compared with low-income women (eTable 15 in Supplement 2). The association between income and severe hypoglycemia in this dataset did not show significant effect modification by obesity, duration of diabetes, or insulin use. An overview of the study design and a summary of the main findings are presented in eFigure 4 in Supplement 1.

## Discussion

Findings through our NHID cohort suggest that risk of severe hypoglycemia increased with lower income. Notably, the risk heightened significantly with even 1 year of medical aid among the extremely poor. However, risk of severe hypoglycemia decreased when income level improved. Similar trends were observed in our UKBB cohort, where the low-income group showed markedly increased risk of severe hypoglycemia. Both cohort analyses consistently suggested that men faced significantly higher risk compared with women.

Well-known risk factors for severe hypoglycemia include advanced age, long duration of diabetes, low body weight, excessive alcohol consumption, insulin and sulfonylurea use, and cognitive dysfunction.^4,15,16,17^ Individuals with low income are likely to exhibit a variety of these known risk factors. Specifically, low-income individuals often require more insulin due to poor diabetes management and are at high risk of neglect due to hypoglycemia unawareness, both stemming from a lack of education on advanced diabetes care.^9^ Additionally, they have higher rates of major comorbidities, including CKD, a significant risk factor for hypoglycemia.^18^ Low-income individuals also face nutritional imbalances, leading to both obesity and a higher risk of being underweight, further increasing their risk of severe hypoglycemia. Previous literature has consistently reported that low-income groups have elevated risk of developing hypoglycemia and severe hypoglycemia. A previous cross-sectional analysis found that food insecurity and exhaustion of food budgets at the end of the month were significantly associated with increased risk of hypoglycemia in low-income groups, with a reduction in hospitalizations due to hypoglycemia being observed during periods when food support through the Supplemental Nutrition Assistance Program was increased.^11,19^ Another study analyzed the risk of severe hypoglycemia by regional level of socioeconomic deprivation and found that residents of the most deprived regions had 41% higher risk of severe hypoglycemia compared with those in less deprived areas.^20^ These findings underscore the importance of addressing socioeconomic disparities in diabetes care to reduce the incidence and severity of hypoglycemia in vulnerable populations. However, studies to date have been limited by cross-sectional design, lack of detailed adjustment for individual-level confounding variables, and failure to account for changes in income, conduct subgroup analyses, or perform mediation analyses.

Income can change over time. Our findings suggest that risk of severe hypoglycemia significantly increased among participants who were medical beneficiaries at least once during the analysis period. However, the risk was markedly reduced among the 170 805 individuals (41.5%) who started out in a lower income group but whose income subsequently increased. This suggests that an increase in income can lower the risk of severe hypoglycemia; moreover, a prolonged period of higher income may further reduce this risk, highlighting financial stability as a major protective factor in diabetes management. Increased income can enhance the care of major risk factors, such as cardiometabolic diseases or CKD, which were significant mediating factors in this study, and can also improve indicators of depression, isolation, and social deprivation.^21,22,23^ From a policy perspective, providing employment support and financial counseling for low-income individuals could help reduce medical and social costs by improving diabetes management and preventing severe hypoglycemia.^24,25^

### Strengths and Limitations

The strength of this study lies in its comprehensive analysis of the association between income and severe hypoglycemia in a large sample of 1.8 million Korean individuals with T2D, with the main findings replicated in 20 000 British individuals from diverse medical, social, and cultural backgrounds. Using long-term data, we measured the outcomes of income changes on severe hypoglycemia over 5 years. First, in the Korean data, the income standard was classified based on the level of health insurance premium payment. There may be classification errors if individuals with higher incomes pay inaccurately reported premiums. Second, the definition of severe hypoglycemia in both cohorts was based on diagnosis codes, which may result in measurement bias. Third, in the UK Biobank data, the income standard was defined based on a questionnaire, which differs from the NHID dataset. Additionally, the definitions and measurement standards for confounding factors varied, making it difficult to directly compare income levels and the risk of severe hypoglycemia between the 2 cohorts. Fourth, the UKBB dataset consists of voluntary participants, who tend to be healthier, have higher health awareness, and belong to higher socioeconomic strata compared with the general population. This introduces a healthy volunteer selection bias, potentially limiting generalizability. Additionally, income categories in the UKBB were derived from self-reported brackets, whereas the NHID dataset classified income based on health insurance premiums. As a result, absolute income levels cannot be directly compared between the 2 cohorts. Instead, our analysis focused on the relative socioeconomic gradient within each dataset, assessing how income disparities within each population were associated with severe hypoglycemia risk. Despite these limitations, previous studies have demonstrated that relative associations observed in the UK Biobank are broadly consistent with findings from population-based cohorts, supporting its utility as a secondary dataset for external validation. Finally, our study primarily relied on NHID data, with the UKBB serving as a supplementary dataset for external validation rather than as a primary source for deriving conclusions. Given the significant difference in sample size between the 2 datasets, our findings should be interpreted with caution, particularly when comparing absolute risk estimates across cohorts.

## Conclusions

The findings of this cohort study provide further evidence that social determinants of health, including social, cultural, and economic factors, were play a critical role in diabetes management. Our findings demonstrated a significant association between income level and the risk of severe hypoglycemia, underscoring the importance of socioeconomic stability in effective disease control. Although both South Korea and the UK provided health care benefits to low-income populations through their national health insurance systems, differences in health literacy, socioeconomic contexts, lifestyle, and environmental factors appeared to modify the association between income and hypoglycemia risk. The heightened vulnerability observed in low-income groups, even among populations traditionally considered at lower risk, highlighted the urgent need for tailored education, early detection, and personalized medical and social interventions to mitigate hypoglycemia risk and its complications. Furthermore, the observed gender disparities suggested the necessity of further research to explore sex-specific influences, including behavioral and social factors, to refine targeted prevention strategies. Addressing these socioeconomic disparities through comprehensive policies could reduce the socioeconomic burden of adverse health outcomes and improve diabetes care and overall health in vulnerable populations.

## Notes

Supplement 1.
eMethods.
**eFigure 1. **Flowchart of Study Population Selection for NHID and UK Biobank Cohorts **eFigure 2. **Study Design and Timeline for NHID (Primary Analysis Dataset) and UK Biobank (Sensitivity Analysis Dataset) **eFigure 3. **Cumulative Incidence of Severe Hypoglycemia Stratified by the Duration Spent in (A) Medical Aid Status, (B) the Lowest Income Quartile (Q1), (C) the Highest Income Quartile (Q4), and (D) the Highest Top 5% Income Group. € Cumulative Incidence of Severe Hypoglycemia Based on Baseline Income Level **eFigure 4.** Graphical Abstract Summarizing the Study Design and Key Findings
eReferences.

Supplement 2. **eTable 1.** Detailed Definitions of Baseline Major Comorbidities and Hypoglycemia Outcome in NHID **eTable 2.** Detailed Definitions of Baseline Major Comorbidities and Hypoglycemia Outcome in UK Biobank **eTable 3. **Summary of Missing Data in the NHID and UK Biobank Cohorts **eTable 4.** Baseline Characteristics According to the Duration in the Medical Aid Receipt Group During Index Period in NHID **eTable 5.** Baseline Characteristics According to the Duration in the 1st Quartile Income Group During Index Period n NHID **eTable 6.** Baseline Characteristics According to the Duration in the Top 5 Percentile Income Group During Index Period in NHID **eTable 7. **Baseline Characteristics According to the Duration in the 4th Quartile Income Group During Index Period in NHID **eTable 8. **Hazard Ratios and 95% CIs for SH Outcomes by Income Group in the Last Year of the Index Period **eTable 9. **Hazard Ratios and 95% CIs for SH Outcomes by Duration in the Income Group During the Index Period **eTable 10. **Hazard Ratios and 95% CIs for SH Outcomes by Changes in Income Group Grom the First Year to the Last Year of the Index Period **eTable 11. **Subgroup Analysis of the Association Between Income Group in the Last Year of the Index Period and SH Outcomes in NHID **eTable 12. **Subgroup Analysis for SH Outcomes by Duration in the Income Group During the Index Period in NHID **eTable 13. **Baseline Characteristics by Income Group in UK Biobank Dataset **eTable 14. **Hazard Ratios and 95% CIs for SH Outcomes by Baseline Income Group in UK Biobank Dataset **eTable 15. **Subgroup Analysis of the Association Between Income Group in the Last Year of the Index Period and SH Outcomes in UK Biobank

Supplement 3.
Data Sharing Statement
