# Supplementary material for: Income and Severe Hypoglycemia in Type 2 Diabetes
Source: JAMA Netw Open. 2025 Jun 2;8(6):e2513293. doi: 10.1001/jamanetworkopen.2025.13293 (PMC12131101; doi:10.1001/jamanetworkopen.2025.13293)
Supplement: Supplement 1. — eMethods. eFigure 1. Flowchart of Study Population Selection for NHID and UK Biobank Cohorts eFigure 2. Study Design and Timeline for NHID (Primary Analysis Dataset) and UK Biobank (Sensitivity Analysis Dataset) eFigure 3. Cumulative Incidence of Severe Hypoglycemia Stratified by the Duration Spent in (A) Medical Aid Status, (B) the Lowest Income Quartile (Q1), (C) the Highest Income Quartile (Q4), and (D) the Highest Top 5% Income Group. € Cumulative Incidence of Severe Hypoglycemia Based on Baseline Income Level eFigure 4. Graphical Abstract Summarizing the Study Design and Key Findings eReferences. [file jamanetwopen-e2513293-s001.pdf]

Supplemental Online Content

Kim M, Han K, Lee K, et al. Income and severe hypoglycemia in type 2 diabetes. *JAMA Netw Open*. 2025;8(6):e2513293. doi:10.1001/jamanetworkopen.2025.13293

eMethods.

eFigure 1. Flowchart of Study Population Selection for NHID and UK Biobank Cohorts

eFigure 2. Study Design and Timeline for NHID (Primary Analysis Dataset) and UK Biobank (Sensitivity Analysis Dataset)

eFigure 3. Cumulative Incidence of Severe Hypoglycemia Stratified by the Duration Spent in (A) Medical Aid Status, (B) the Lowest Income Quartile (Q1), (C) the Highest Income Quartile (Q4), and (D) the Highest Top 5% Income Group. € Cumulative Incidence of Severe Hypoglycemia Based on Baseline Income Level

eFigure 4. Graphical Abstract Summarizing the Study Design and Key Findings

eReferences.

This supplemental material has been provided by the authors to give readers additional information about their work.

© 2025 Kim M et al. *JAMA Network Open*.

eMethods

Data sources and demographic information

The data were collected from the National Health Information Database (NHID) of South Korea and the UK Biobank database. The National Health Insurance System (NHIS), a single-payer healthcare system managed by the South Korean government, operates a mandatory national health insurance program that covers 97% of the South Korean population. The NHID does not explicitly collect race or ethnicity data. However, previous genomic studies indicate that the Korean population is predominantly of East Asian descent,[1] supporting the assumption that NHID participants primarily belong to the East Asian ethnic group. The NHID, maintained by the NHIS, includes demographic information, diagnostic history, prescription records, medical procedures, inpatient and outpatient care data, and mortality records. Additionally, all regional and workplace subscribers aged 20 years or older, as well as their dependents, are required to undergo health examinations annually or at least once every two years. In particular, non-office workers enrolled in workplace insurance must undergo mandatory annual health examinations. Meanwhile, the UK Biobank has collected comprehensive health data from approximately 500,000 middle-aged participants (aged 40–70 years) across the United Kingdom between 2006 and 2010, encompassing individuals from diverse ethnic backgrounds. Most health outcomes followed up continuously. In the UK Biobank, race and ethnicity were self-reported by participants during the baseline assessment using a standardized questionnaire (Data-Field 21000). Participants selected their ethnic background from predefined categories, including White, Mixed, Asian, Black, Chinese, and Other ethnic groups, based on the UK Office for National Statistics classification.

Income assessment and classification

In South Korea, NHIS determines health insurance premiums according to monthly household income, using it as an indicator of income level. For employed individuals, premiums are based on salaries across all occupational categories, while for self-employed individuals and others without fixed salaries, contributions are determined based on reported income, assets, and other socioeconomic factors. Low-income individuals who qualify for Medical Aid are classified as the lowest income group. As a result, the NHID can estimate income levels from health insurance premiums instead of providing actual household income data. If an individual's income drops to meet the medical benefit standards in the previous year, they become eligible for medical benefits the following year. Conversely, if their income rises above the threshold, they switch to the NHIS.

Missing data management

For the NHID cohort, a complete case analysis was applied, excluding individuals with missing data for any covariates from all analyses. For the UK Biobank cohort, an available-case analysis was used, excluding individuals only from analyses requiring the missing covariate.

© 2025 Kim M et al. *JAMA Network Open*.

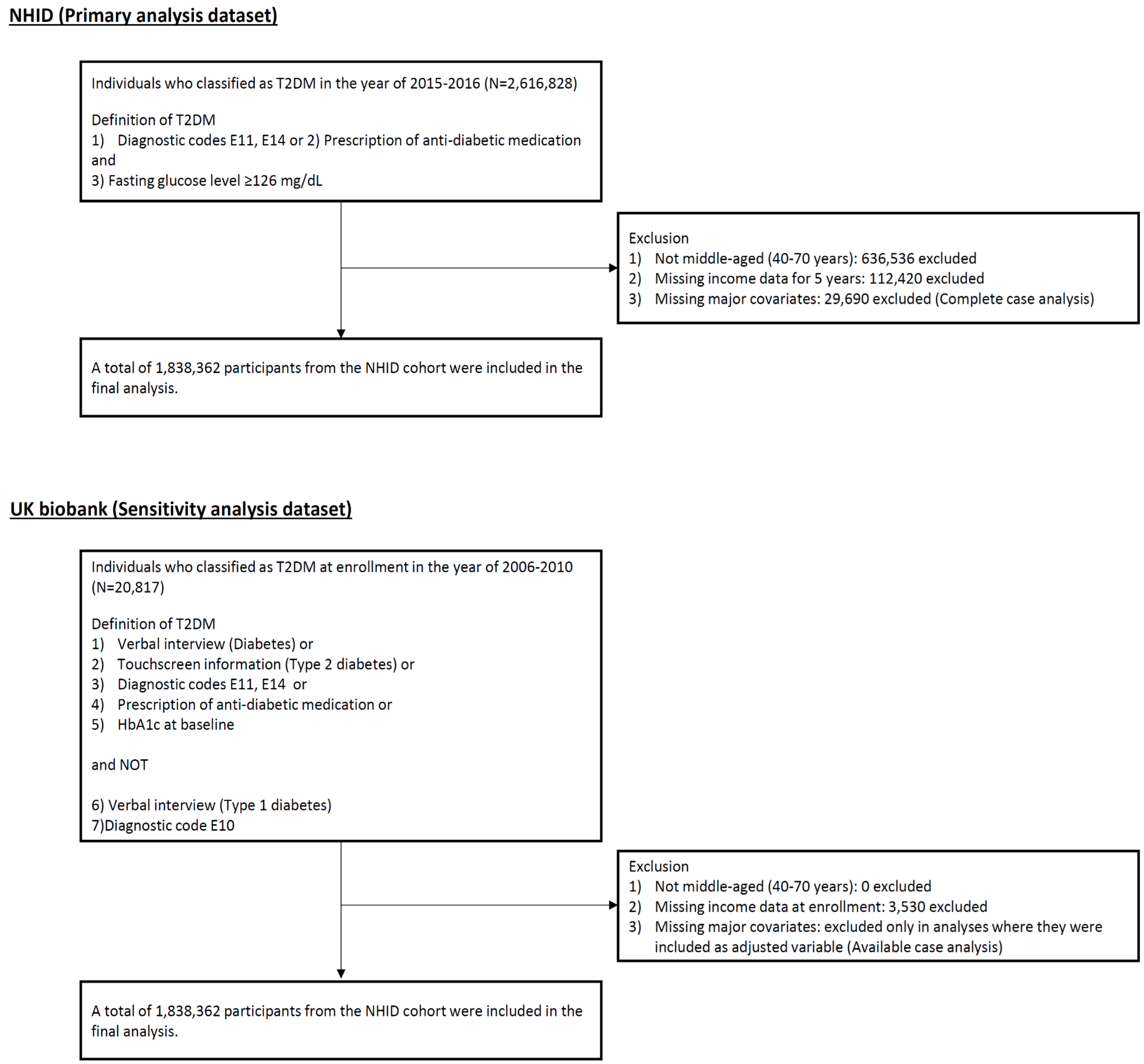

eFigure 1. Flowchart of Study Population Selection for NHID and UK Biobank Cohorts

© 2025 Kim M et al. *JAMA Network Open*.

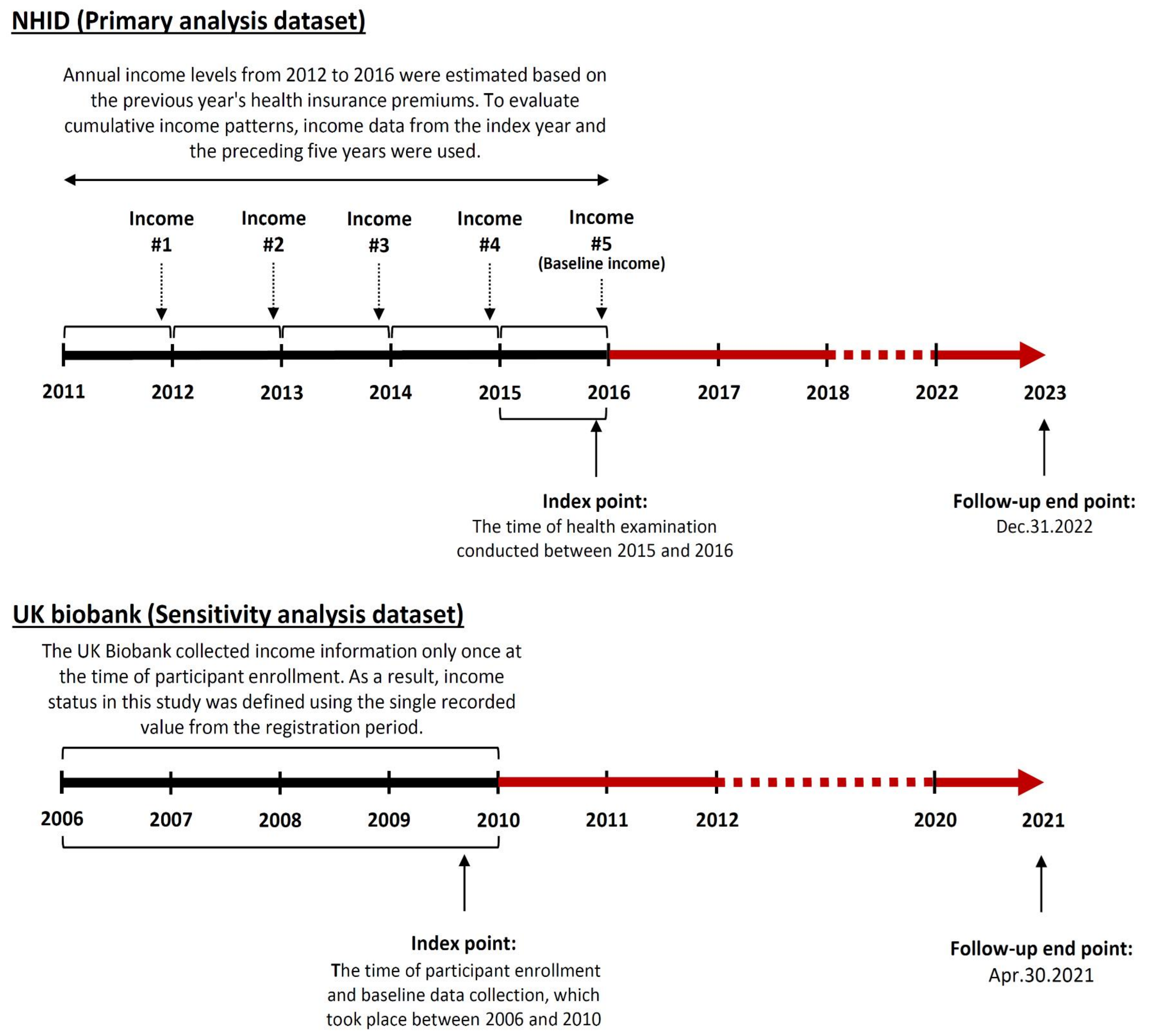

eFigure 2. Study Design and Timeline for NHID (Primary Analysis Dataset) and UK Biobank (Sensitivity Analysis Dataset)

© 2025 Kim M et al. *JAMA Network Open*.

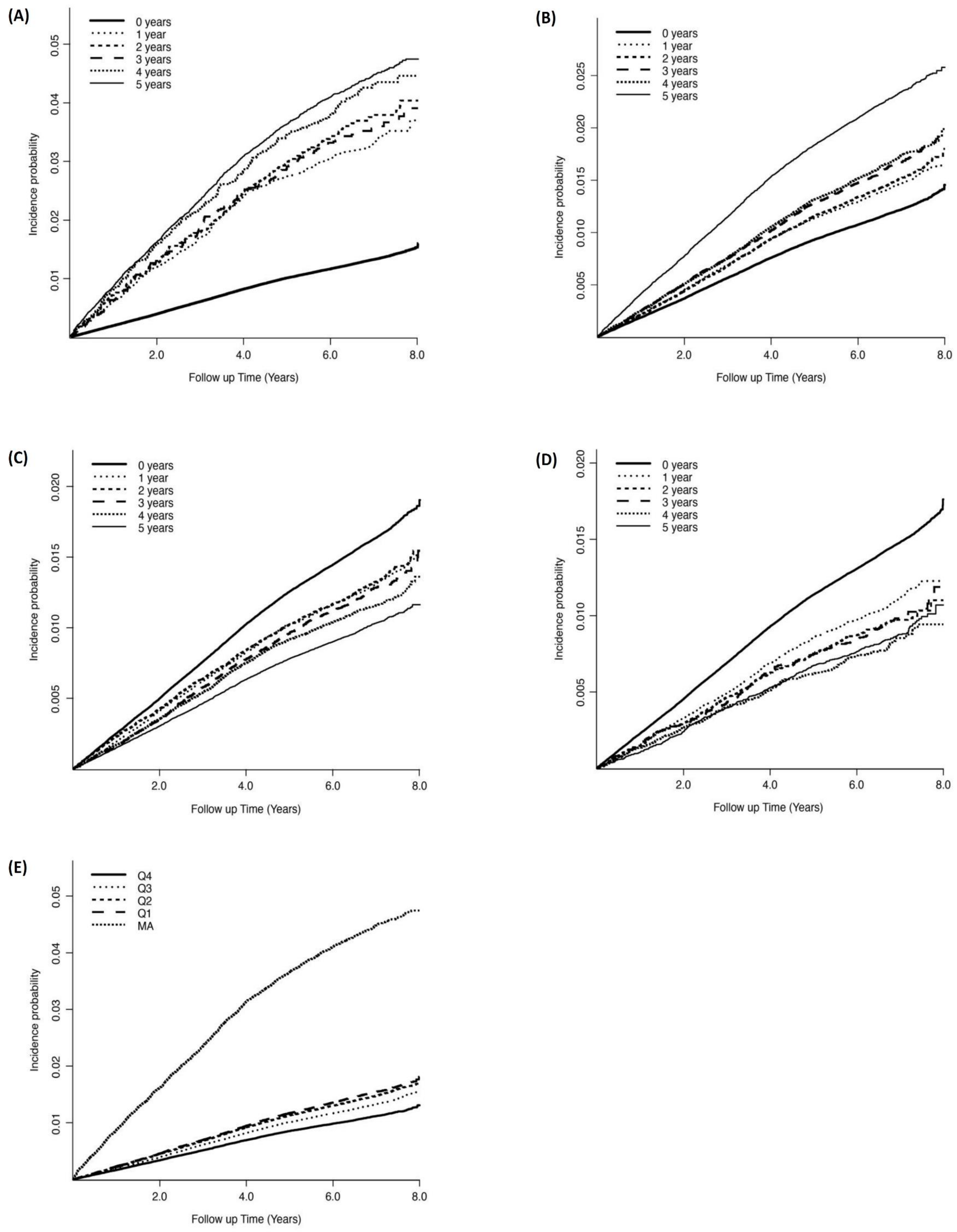

eFigure 3. Cumulative Incidence of Severe Hypoglycemia Stratified by the Duration Spent in (A) Medical Aid Status, (B) the Lowest Income Quartile (Q1), (C) the Highest Income Quartile (Q4), and (D) the Highest Top 5% Income Group. (E) Cumulative Incidence of Severe Hypoglycemia Based on Baseline Income Level

© 2025 Kim M et al. *JAMA Network Open*.

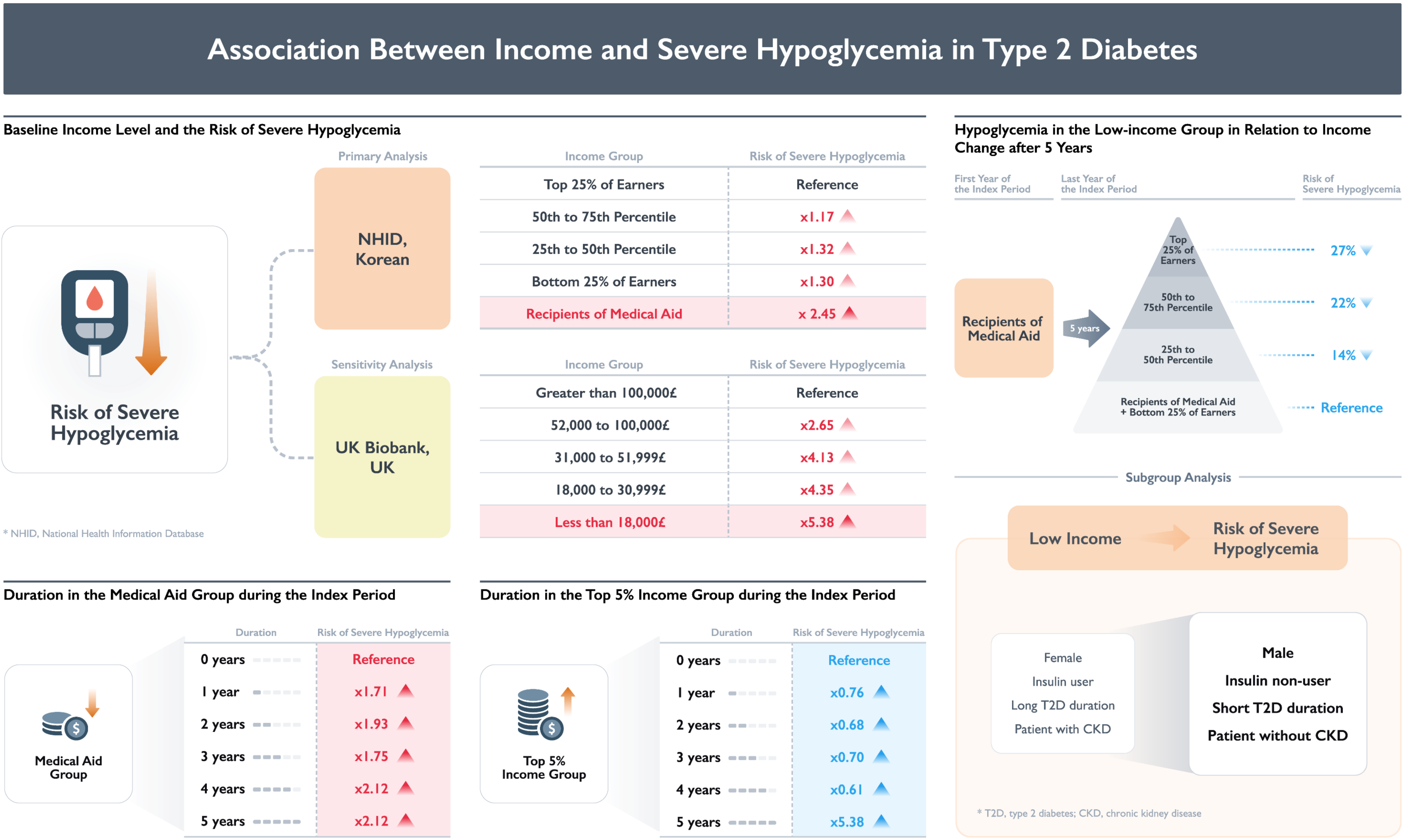

eFigure 4. Graphical Abstract Summarizing the Study Design and Key Findings

*This figure was created by a third party specifically for this manuscript and is used here with permission.

© 2025 Kim M et al. *JAMA Network Open.*

eReferences

1. Kim J, Jeon S, Choi JP, et al. The Origin and Composition of Korean Ethnicity Analyzed by Ancient and Present-Day Genome Sequences. *Genome Biol Evol*. May 1 2020;12(5):553-565. doi:10.1093/gbe/evaa062

© 2025 Kim M et al. *JAMA Network Open*.
