# Supplementary material for: Income and Severe Hypoglycemia in Type 2 Diabetes
Source: JAMA Netw Open. 2025 Jun 2;8(6):e2513293. doi: 10.1001/jamanetworkopen.2025.13293 (PMC12131101; doi:10.1001/jamanetworkopen.2025.13293)
Supplement: Supplement 3. — Data Sharing Statement [file jamanetwopen-e2513293-s003.pdf]

Data Sharing Statement

Kim. Income and Severe Hypoglycemia in Type 2 Diabetes. *JAMA Netw Open*. Published June 02, 2025. doi:10.1001/jamanetworkopen.2025.13293

Data
Data available: No
